# Analgesic Effect of Electroacupuncture in a Mouse Fibromyalgia Model: Roles of TRPV1, TRPV4, and pERK

**DOI:** 10.1371/journal.pone.0128037

**Published:** 2015-06-04

**Authors:** Jaung-Geng Lin, Ching-Liang Hsieh, Yi-Wen Lin

**Affiliations:** 1 College of Chinese Medicine, School of Chinese Medicine, China Medical University, Taichung 40402, Taiwan; 2 College of Chinese Medicine, Graduate Institute of Integrative Medicine, China Medical University, Taichung 40402, Taiwan; 3 China Medical University Hospital, Department of Chinese Medicine, Taichung, 40402, Taiwan; 4 Research Center for Chinese Medicine & Acupuncture, China Medical University, Taichung 40402, Taiwan; 5 College of Chinese Medicine, Graduate Institute of Acupuncture Science, China Medical University, Taichung 40402, Taiwan; University of California, Los Angeles, UNITED STATES

## Abstract

Fibromyalgia (FM) is among the most common chronic pain syndromes encountered in clinical practice, but there is limited understanding of FM pathogenesis. We examined the contribution of transient receptor potential vanilloid 1 (TRPV1) and TRPV4 channels to chronic pain in the repeated acid injection mouse model of FM and the potential therapeutic efficacy of electroacupuncture. Electroacupuncture (EA) at the bilateral Zusanli (ST36) acupoint reduced the long-lasting mechanical hyperalgesia induced by repeated acid saline (pH 4) injection in mouse hindpaw. Isolated L5 dorsal root ganglion (DRG) neurons from FM model mice (FM group) were hyperexcitable, an effect reversed by EA pretreatment (FM + EA group). The increase in mechanical hyperalgesia was also accompanied by upregulation of TRPV1 expression and phosphoactivation of extracellular signal regulated kinase (pERK) in the DRG, whereas DRG expression levels of TRPV4, p-p38, and p-JNK were unaltered. Blockade of TRPV1, which was achieved using TRPV1 knockout mice or via antagonist injection, and pERK suppressed development of FM-like pain. Both TRPV1 and TRPV4 protein expression levels were increased in the spinal cord (SC) of model mice, and EA at the ST36 acupoint decreased overexpression. This study strongly suggests that DRG TRPV1 overexpression and pERK signaling, as well as SC TRPV1 and TRPV4 overexpression, mediate hyperalgesia in a mouse FM pain model. The therapeutic efficacy of EA may result from the reversal of these changes in pain transmission pathways.

## Introduction

Activation of acid-sensitive ion channels may contribute to the pain of fibromyalgia (FM) [1–4]. Indeed, acidosis from lactate accumulation is a common trigger for muscle pain [5,6]; FM is strongly associated with acid-sensing ion channel 3 (ASIC3) [4]. Repeated acid injection can reliably produce an FM-like condition in animals and this animal model may be valuable for elucidating the pathogenesis and improving treatment for chronic muscle pain in FM [1–4,7]. Substance P (SP) [3], the Ca_v_3.2 T-type Ca^2+^ channel (Ca_v_3.2) [1], and phosphorylated extracellular signals regulated kinase (pERK) in either the peripheral nervous system (PNS) or central nervous system (CNS) have been implicated in physiological pain transmission and FM-associated pain [8,9]. The transient receptor potential vanilloid (TRPV) family of channels is also involved in pain signaling in both peripheral and central nervous systems, and thus may be altered in chronic pain conditions. However, the contribution of these channels to FM is unclear. The TRPV family comprises six subtypes, TRPV1-6 [10–12]; changes in the expression of TRPV1 and TRPV4 have been associated with both mechanical and thermal hyperalgesia [13,14]. The TRPV1 channel is commonly regarded as a receptor of inflammatory and thermal pain in response to noxious heat (>43°C) [15,16]. Recently, TRPV1 was shown to be highly expressed in dorsal root ganglion (DRG) neurons and to contribute to cancer pain [17]. TRPV4 gene knockdown reduces responsively to osmotic stimuli [18,19]. The TRPV4 channel is expressed in several tissues (liver, kidney, heart, and airway epithelia) where it is involved in mechanoregulation [18,20]. TRPV4 is also reported to mediate several kinds of pain, such as mechanical hyperalgesia and pain associated with diabetes and acquired immune deficiency syndrome therapy [21,22]. Grant et al. demonstrated that inflammation could activate second messengers, including phospholipase Cβ (PLCβ), protein kinase A (PKA), and PKC, which further activate TRPV4, leading to the release of pain transmitters CGRP and SP in the spinal dorsal horn [23].

Acupuncture is highly effective for treating certain pain symptoms [24–28]. Pain reduction by acupuncture is blocked by procaine injection, indicating that the analgesic effects may be mediated by release of endogenous opiates [27]. Goldman *et al*. suggested that the analgesic effect of acupuncture was mediated in part by the release of adenosine triphosphate (ATP), which is further metabolized to adenosine by prostatic acid phosphatase (PAP). In mice, adenosine then activates A1 receptors (A1R) to block transmission of inflammatory and neuropathic pain [26]. A recent study found that injecting an A1R agonist into the Weizhong acupoint had a short-term antinociceptive effect and that the peripheral injection of PAP into this acupoint produced a long-lasting analgesic effect on chronic inflammatory and neuropathic pain [29].

We previously suggested that TRPV1 and TRPV4 could also contribute to electroacupuncture (EA)-mediated analgesia in a mouse inflammatory pain model [25]. Here we demonstrate that EA suppresses mechanical hyperalgesia in the acid-induced mouse FM model, possibly by reducing hyperalgesia-associated DRG neuron hyperexcitability, TRPV1 overexpression, and activation of ERK signaling pathways as well as TRPV1 and TRPV4 overexpression in the spinal cord (SC). Thus, EA may reduce pain in this model through peripheral and central effects.

## Materials and Methods

### Animals and EA pretreatment

In total, 120 adult C57/B6 (BioLASCO Taiwan Co., Ltd) mice aged 8 to 12 weeks were used in this study. After their arrival, the mice were maintained using a 12 h light:dark cycle and provided with sufficient food and water. To minimize their suffering, at the appropriate point in the experiment, mice were anesthetized and killed with isoflurane. The usage of these animals was approved by the *Institute of Animal Care and Use Committee of China Medical University (permit No*. *101-116-N)*, *Taiwan following the Guide for the use of Laboratory Animals* (National Academy Press). We use EA on mice by inserting a stainless steel acupuncture needles (1.5” inch, 32G, YU KUANG, Taiwan) into the ST36 acupoint at a depth of 3–4 mm. Square pulses electrical stimulation were delivered for 15 min with a duration of 100 μs and 2 Hz in frequency generated from the stimulator. The stimulation amplitude was 1 mA. EA was administered immediately after the second injection of acid saline and performed at the same time every day (i.e., 1:00–4:00 PM). The von Frey assessment was conducted 1 h after EA treatment. The similar protocol was given to ST36 acupoint without electrical stimulation (without De-qi) as the sham control group.

FM induction, pharmacological injection, and animal behavior of mechanical hyperalgesia

We injected 20 μL of pH 4.0 saline into the gastrocnemius muscle (GM) while the mice were anesthetized with isoflurane (1%). A second acid injection was delivered 5 days later to induce the mouse FM model with or without 10 μL capsazepine (1 nM), U0126 (1 μg in 10% DMSO) injected in ST36 acupoint. FM was also induced in TRPV1 knockout mice to investigate its role in this mouse model. Mechanical sensitivities were tested 8 days after the FM model was first induced and 1 h after EA manipulation or pharmacological injection. Mice were adapted to the new environment for at least 30 min and the stimuli were applied only when the animals were not sleeping or grooming. All experiments were performed at room temperature (approximately 25°C) and Mechanical hyperalgesia was examined by applying a 0.2-mN von Frey filament to the plantar of hind paws. Mice were calm down to the new environment for at least 30 min. The mechanical hyperalgesia of the hindpaw was measured before modeling, and 4 h, 1 day, 5 day, 6 day, and 8 day after acid saline injection.

### Immunohistochemistry

L3-L5 DRG and lumbar SC neurons were immediately dissected and post-fixed with 4% paraformaldehyde. For TRPV1 and TRPV4 protein analysis, DRG and SC samples were dissected 8 days after the first acid injection and stored at −80°C. For pERK analysis, samples were collected 15 min after the second acid injection. Post-fixed tissues were then placed in 30% sucrose overnight for cryoprotection. The DRGs were then embedded in OCT and rapidly frozen at −20°C. Frozen sections were cut in a 12-μm thick on a cryostat. Samples were next incubated with blocking solution containing 3% BSA, 0.1% Triton X-100, and 0.02% sodium azide in PBS for 120 min at room temperature. After blocking, DRGs were incubated with primary antibodies prepared in blocking solution at 4°C overnight against TRPV1 (1:1000, Alomone), TRPV4 (1:1000, Alomone), and pERK (1:1000, Alomone). The secondary antibodies were goat anti-rabbit 488 (Molecular Probes, Carlsbad, CA, USA) and goat anti-mouse 594 (Molecular Probes, Carlsbad, CA, USA). Slides were visualized by use of fluorescence-conjugated secondary antibodies and mounted on coverslips. The stained DRG slices were sealed under the coverslips, and then examined for the presence of immune-positive DRG neurons using an epi-fluorescent microscope (Olympus, BX-51, Japan) with a 40 × numerical aperture (NA = 1.4) objective. Furthermore, all images were analyzed using NIH ImageJ software (Bethesda, MD, USA).

### Western blot analysis

L3-L5 DRG and lumbar SC neurons were immediately excised to extract proteins. For TRPV1 and TRPV4 protein analysis, DRG and SC samples were dissected 8 days after the first acid injection and stored at −80°C. For pERK, pp38, and pJNK analysis, samples were collected 15 min after the second acid injection. Total proteins were prepared by homogenized sample in lysis buffer containing 50 mM Tris-HCl pH 7.4, 250 mM NaCl, 1% NP-40, 5 mM EDTA, 50 mM NaF, 1 mM Na3VO4, 0.02% NaN3 and 1× protease inhibitor cocktail (AMRESCO). The extracted proteins (30μg per sample assessed by BCA protein assay) were subjected to 8% SDS-Tris glycine gel electrophoresis and transferred to a PVDF membrane. The membrane was blocked with 5% nonfat milk in TBS-T buffer (10 mM Tris pH 7.5, 100 mM NaCl, 0.1% Tween 20), incubated with anti-TRPV1, anti-TRPV4, anti-pERK, anti-pp38, and anti-pJNK antibody (1:1000, Alomone) in TBS-T with 1% bovine serum albumin, and incubated for 1 hour at room temperature. Peroxidase-conjugated anti-rabbit antibody (1:5000) was used as a secondary antibody. The bands were visualized by an enhanced chemiluminescencent substrate kit (PIERCE) with LAS-3000 Fujifilm (Fuji Photo Film Co. Ltd). Where applicable, the image intensities of specific bands were quantified with NIH ImageJ software (Bethesda, MD, USA).

### DRG primary cultures and whole-cell patch-clamp recording

C57/B6 mice aged 8–12 weeks were sacrificed by using CO2 to minimize their suffering. L3–L5 DRG neurons were dissected and placed in a tube containing DMEM and then transferred to DMEM with type I collagenase (0.125%, 120 min) for digestion at incubator at 37°C. Neurons were then plated on poly-L-lysine-coated coverslips. All recordings were completed within 24 hours after plating. Glass pipettes (Warner Products 64–0792) were prepared (1–5 MΩ) with use of a vertical puller (NARISHIGE PC-10). Whole-cell recordings involved use of an Axopatch MultiClamp 700B (Axon Instruments). Stimuli were controlled and digital records captured with use of Signal 3.0 software and a CED1401 converter (Cambridge Electronic Design). Cells with a membrane potential more positive than −40 mV were not accepted. The bridge was balanced in current clamping recording. Recording cells were superfused in artificial cerebrospinal fluid (ACSF) containing (in mM) 130 NaCl, 5 KCl, 1 MgCl2, 2 CaCl2, 10 glucose, and 20 HEPES, adjusted to pH 7.4 with NaOH. ACSF solutions were applied by use of gravity. The recording electrodes were filled with (in mM) 100 KCl, 2 Na2-ATP, 0.3 Na3-GTP, 10 EGTA, 5 MgCl2, and 40 HEPES, adjusted to pH 7.4 with KOH. Osmolarity was approximately 300–310 mOsm. The action potential (AP) parameters were determined using a current clamp mode. First, the resting membrane potential, rise time, fall time, and AHP duration (80% recovery to baseline) were measured from a single AP elicited by a 1-ms 2-nA current step. Subsequently, a 50-ms current step was used to determine AP threshold.

### Statistical analysis

All statistic data are presented as the mean ± standard error. Statistical significance between control, FM, and EA group was tested using the ANOVA test, followed by a post hoc Turkey’s test (*p* < 0.05 was considered statistically significant).

## Results

### Low frequency EA attenuates mechanical hyperalgesia induced by repeated acid injection

**Fig 1:**
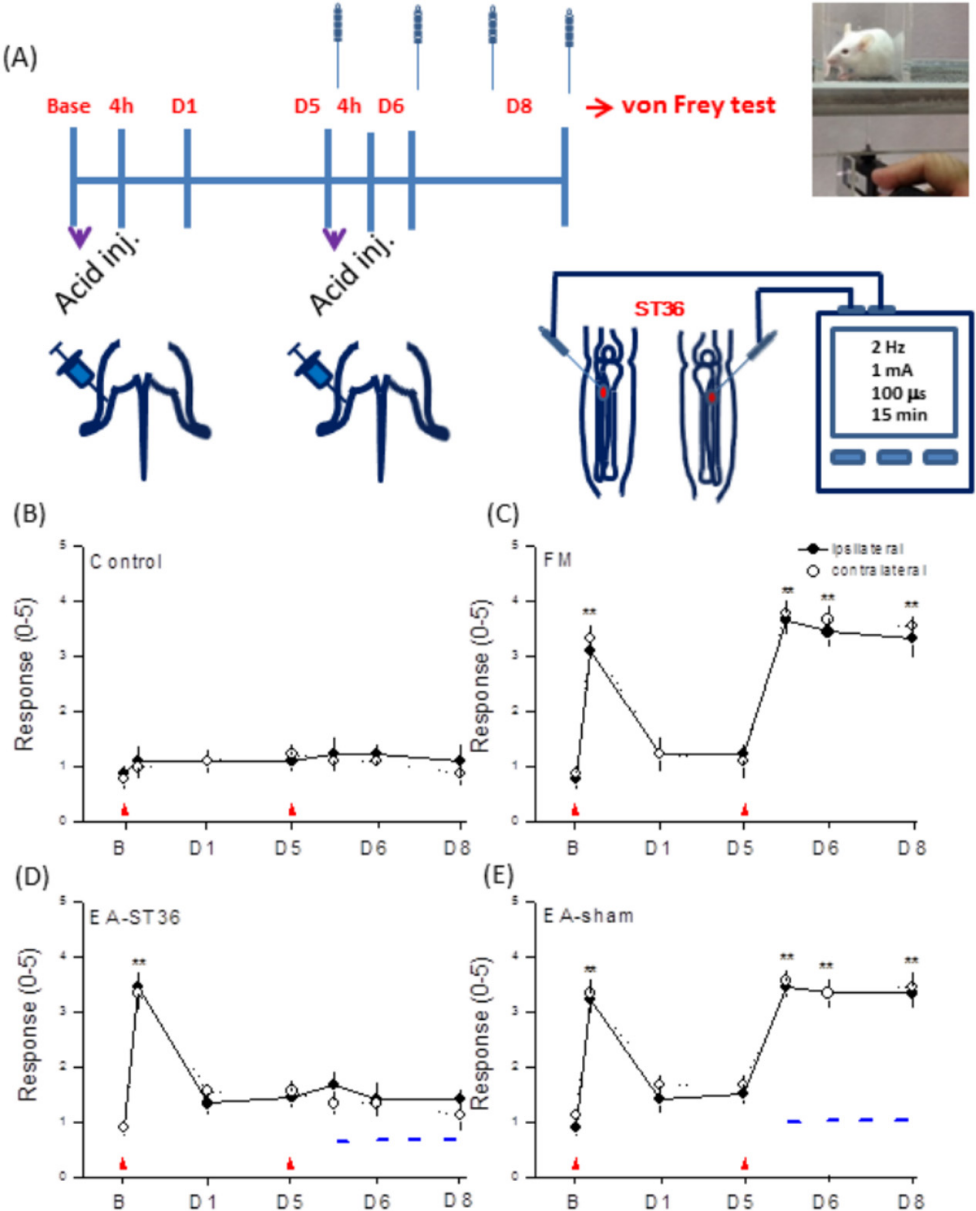
Electroacupuncture (EA) attenuated mechanical hyperalgesia induced by repeated intramuscular acid saline injection (fibromyalgia model, FM) as measured by von Frey filaments. (A) Mechanical responses of saline-injected control mice. (B) Mechanical responses of FM group mice. (C) Mechanical responses of FM mice pretreated with EA (FM + EA group). (D) Mechanical responses of FM mice pretreated by sham EA. Mice were tested before injection (baseline, B), 4 hours after injection, day 1 (D1), day 5 (D5), day 6 (D6), and day 8 (D8). Red arrowheads indicate acid injection; Blue dashes indicate EA. **p < 0.01 compared to baseline (n = 9 mice per group).

To test if EA could reverse acid-induced mechanical hyperalgesia, we compared responses to von Frey filaments at baseline, and at D1, D5, D6, and D8 post-injection among control, FM model, and FM + EA groups. Intramuscular injection of pH 7.0 normal saline did not initiate mechanical hyperalgesia (baseline = 0.89 ± 0.11 g; first injection = 1.11 ± 0.24 g; n = 9; *p* > 0.05; second injection: D5 = 1.11 ± 0.17 g; D6 = 1.22 ± 0.18 g; D8 = 1.11 ± 0.29 g, n = 9; *p* > 0.05; Fig 1A, black circles). Similar results were observed at the contralateral site (baseline = 0.78 ± 0.15 g; D1 = 1.0 ± 0.17 g; D5 = 1.33 ± 0.18 g; D6 = 1.11 ± 0.11 g; D8 = 0.89 ± 0.20 g, n = 9; *p* > 0.05; Fig 1A, white circles). In contrast, a single intramuscular injection of acidic saline (pH 4) evoked mechanical hyperalgesia (baseline = 0.78 ± 0.15 g; first acid injection = 3.11 ± 0.11 g; n = 9; *p* < 0.01; Fig 1B, black circles). However, this mechanical hyperalgesia declined after one day (2.22 ± 0.22 g; n = 9; *p* < 0.05; Fig 1B, black circles). A second acid injection administered 5 days after the first induced mechanical hyperalgesia that was maintained for 8 days (D5 = 3.67 ± 0.24; D6 = 3.44 ± 0.24 g; D8 = 3.33 ± 0.33 g; n = 9, *p* < 0.01; Fig 1B, black circles,). Moreover, a similar pattern was obtained at the contralateral site (baseline = 0.89 ± 0.11 g; first acid injection = 3.33 ± 0.24 g; D5 = 3.78 ± 0.22 g; D6 = 3.67 ± 0.24 g; D8 = 3.56 ± 0.18, n = 9, *p* < 0.01; Fig 1A, white circles), suggesting central sensitization. In some FM model mice, low-frequency EA was delivered at the ST36 acupoint once daily on D5, D6, D7, and D8 prior to mechanosensitivity tests. This treatment (FM + EA group) reliably decreased mechanical hyperalgesia (baseline = 0.89 ± 0.11 g; D5 = 2.56 ± 0.24 g; D6 = 2.44 ± 0.29 g; D8 = 2.33 ± 0.17, n = 9, *p* < 0.05 compared to the FM group; Fig 1C, black circles), while sham EA had no effect (baseline = 0.89 ± 0.11 g; D5 = 3.44 ± 0.18 g; D6 = 3.33 ± 0.24 g; D8 = 3.33 ± 0.24, n = 8, *p* < 0.01; Fig 1D, black circles). These results indicate that EA at the ST36 acupoint (but not needle penetration alone) can ameliorate mechanical hyperalgesia in this FM model.

### FM modeling altered the biophysical properties of DRG neurons

**Fig 2:**
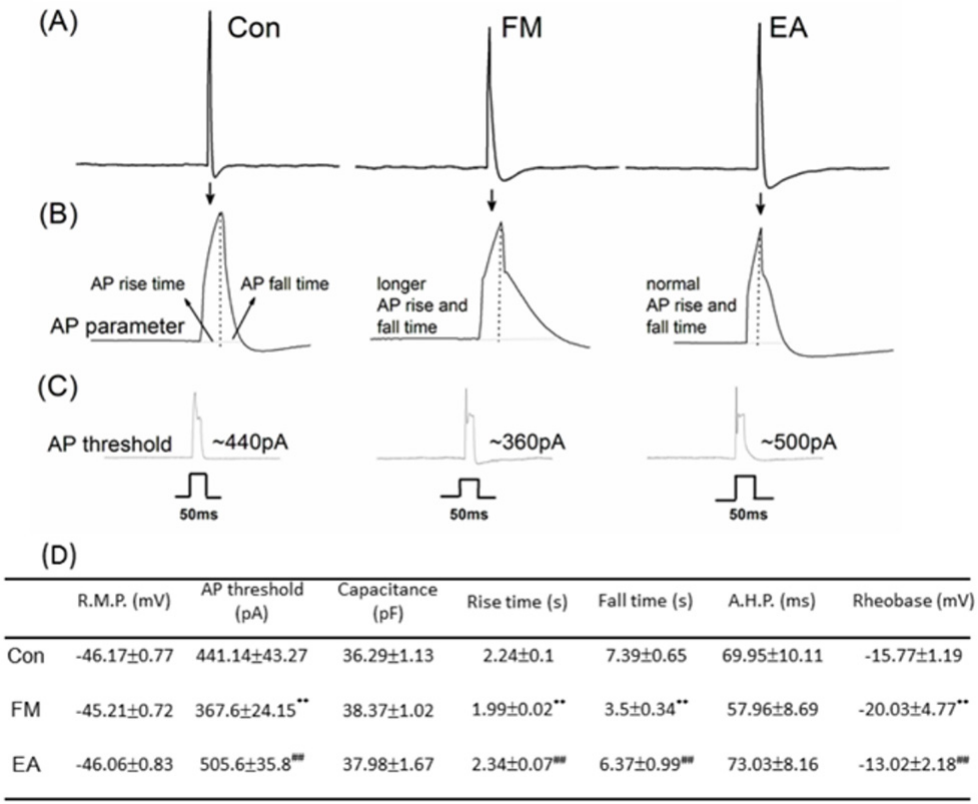
Electrophysiological properties of L3-5 dorsal root ganglion (DRG) neurons from Con, FM, and EA groups. (A) The action potential (AP) threshold was lower in the FM group than the control group. EA reduced neuronal excitation by increasing AP threshold, reversing the effect of acid injection. (B) FM induction decreased both AP rise and fall times compared to controls, effects also reversed by AE. (C) There were no significant group differences in AP amplitude and afterhyperpolarization (AHP) duration. (D) Data summary and statistical analyses.

We next examined changes in the electrophysiological properties of isolated DRG neurons from control, FM, and FM + EA group mice using whole-cell patch clamping. Membrane potential and capacitance did not differ among the 3 groups (Fig 2A). However, membrane excitability was higher in DRG neurons isolated from FM mice on day 8 compared to the other two groups. The AP threshold and rheobase were lower in the FM group (367.6 ± 24.15 pA and −20.03 ± 4.77 mV, *p* < 0.01, n = 25 cells; Fig 2B) compared to controls, an effect that was reversed by EA (Fig 2B, 505.6 ± 35.8 pA and -13.02 ± 2.18 mV, respectively, *p* < 0.01 compared to the FM group, n = 25). Furthermore, AP rise and fall times were significantly shorter in DRG neurons from FM group mice (1.99 ± 0.02 ms and 3.5 ± 0.34 ms respectively, *p* < 0.01, n = 25; Fig 2B) compared to controls, and again these effects were reversed by EA (2.34 ± 0.07 ms and 6.37 ± 0.99 ms, respectively, *p* < 0.01, n = 25; Fig 2B). There were no significant group differences in AP amplitude and afterhyperpolarization (AHP) duration (Fig 2C). Summary results with statistical analyses are presented in Fig 2D.

### FM modeling altered TRPV1 but not TRPV4 receptor expression in DRG neurons

**Fig 3:**
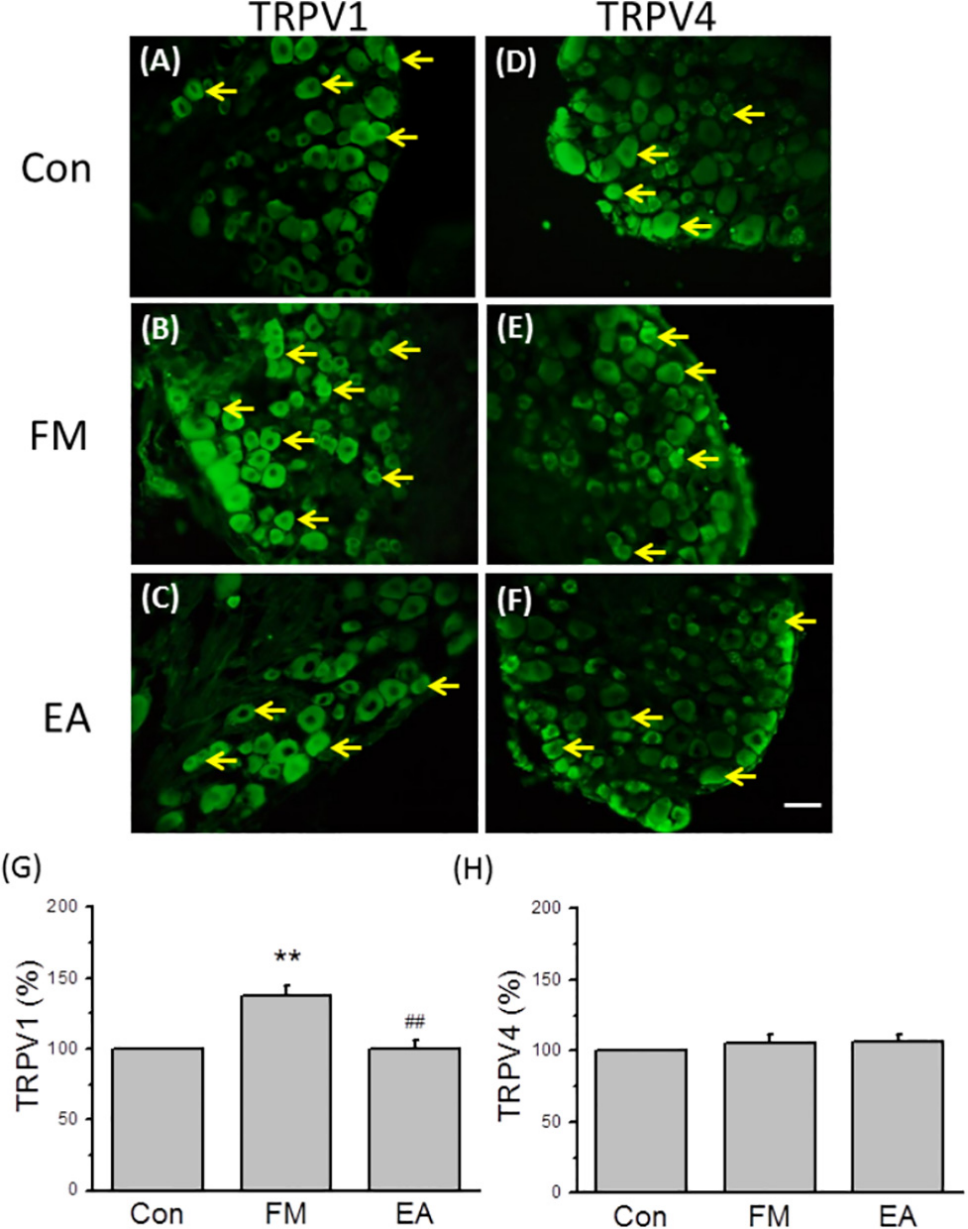
Upregulation of TRPV1 immunohistochemical expression in L3-5 DRG neurons from FM model mice and reversal by EA. (A-C) Immunohistochemical staining showing TRPV1-positive cells (green) in control (A), FM (B), and EA groups (C). (D-F) Immunohistochemical staining showing TRPV4-positive neurons (green) in control (D), FM (E), and EA groups (F). (G, H) Proportions of immunopositive neurons. Con = Control; FM = acid induced fibromyalgia pain; EA = FM pain with electroacupuncture. DRG = dorsal root ganglion. Arrows identify immunopositive neurons. Scale bar = 50 μm.

TRPV1 and TRPV4 receptors contribute to inflammatory pain and can be regulated by EA [25]. We next examined if TRPV1 and TRPV4 were altered by FM modeling and EA manipulation using immunohistochemistry staining and Western blotting. TRPV1-immunoreactive (IR) cells were widely distributed in the DRG (Fig 3A). The number of TRPV1-IR neurons was higher in the FM group than the control group on day 8, while numbers were similar to control in the FM + EA group (Fig 3B and 3C). In contrast to TRPV1, expression of TRPV4 receptors did not appear altered in DRG from FM and FM + EA group mice (Fig 3D–3F). These results suggest that TRPV1 upregulation may contribute to hyperalgesia, while reversal of this upregulation may account for the analgesic effects of EA.

**Fig 4:**
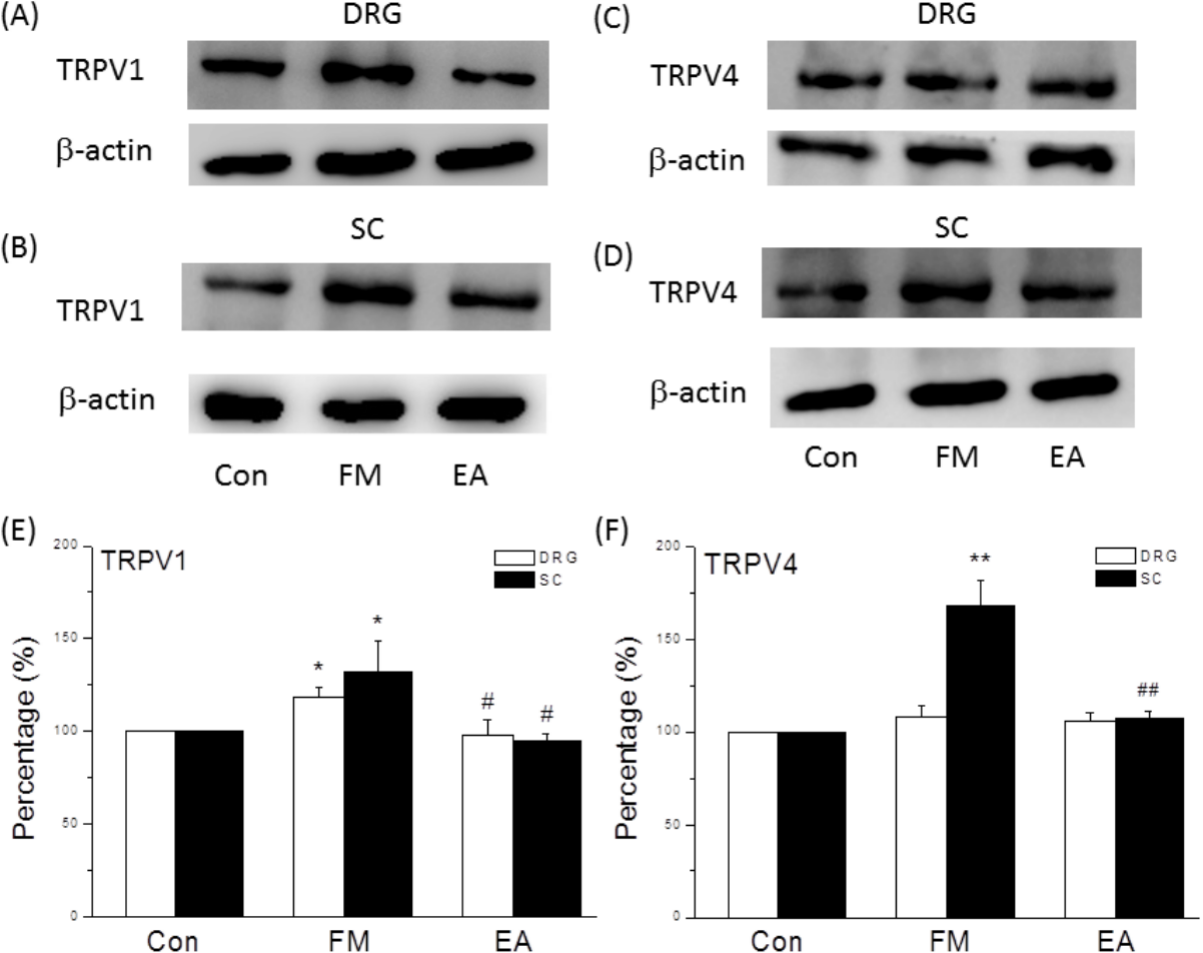
Upregulation of TRPV1 protein expression in DRG neurons from FM mice, upregulation of both TRPV1 and TRPV4 in lumbar spinal cord (SC) of FM mice, and reversal of overexpression by EA. (A) Western blots of DRG lysates showing TRPV1 upregulation in FM mice compared to control mice and reversal by EA. (B) Upregulation of TRPV1 protein in lysates from SC. (C) TRPV4 expression levels were unaltered in the DRG of Con, FM, and EA groups. (D) Upregulation of TRPV4 in the SC of FM mice and reversal by EA. β-actin was used as the internal control. (E, F) Proportions of immunopositive neurons. Con = Control; FM = acid induced fibromyalgia pain; EA = electroacupuncture. DRG = dorsal root ganglion. SC = spinal cord.

Similar results were obtained using Western blot analyses. Expression of TRPV1 proteins was higher in the DRG of FM mice compared to controls (118.40 ± 5.23%, n = 6, *p* < 0.05; Fig 4A), and this overexpression was reversed by EA (Fig 4A, 98.09 ± 8.01%, n = 6, *p* < 0.05 compared to the FM group). TRPV1 expression was also unregulated in the spinal cord after FM modeling and, as in the DRG, reversed by EA (FM: 132.08 ± 16.41%; FM + EA: 94.47 ± 4.07%, n = 6, *p* < 0.05; Fig 4B). Notably, TRPV4 protein level was unchanged in DRG neurons after FM and EA treatment (FM: 107.79 ± 6.04%, FM + EA: 105.81 ± 4.43%, n = 6, *p* > 0.05; Fig 4C). In the spinal cord, however, TRPV4 was potentiated in the FM group (Fig 4D, 167.52 ± 14.37%, n = 6, *p* < 0.05), and this overexpression was reversed by EA (107.19 ± 4.06%, n = 6, *p* < 0.05 compared to the FM group; Fig 4D). Densitometric analyses are shown in Fig 4E and 4F. These results suggest that TRPV1 upregulation may be involved in hyperalgesia at the peripheral level while both TRPV1 and TRPV4 overexpression may contribute to central sensitization. Moreover, the analgesic effects of EA appear to be mediated by reversal of TRPV1 and/or TRPV4 overexpression.

### EA decreases ERK phosphoactivation in the DRG

**Fig 5:**
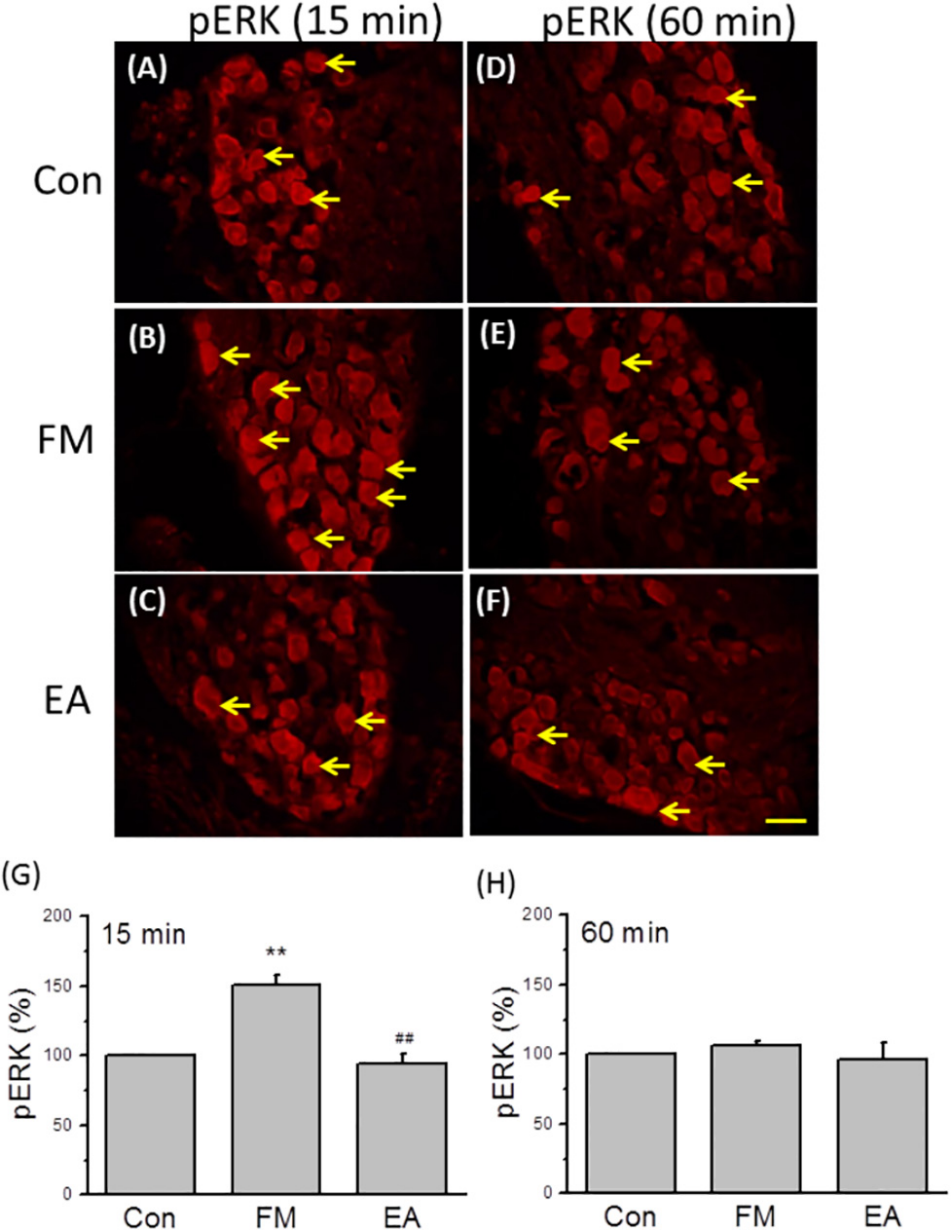
Protein expression of pERK in DRG neurons from Con, FM, and EA groups. (A-C) Immunohistochemical staining showing pERK-reactive cells in the L3-5 DRG of control (A), FM (B), and EA mice (C) 15 min after intramuscular injection of normal saline (control) or acid saline (FM and EA groups). (D-F) Immunohistochemical staining showing pERK-reactive neurons at 60 min after injection (red). (G, H) Proportions of immunopositive neurons. Con = Control; FM = acid induced fibromyalgia pain; EA = electroacupuncture. DRG = dorsal root ganglion. Arrows mean immuno-positive neurons. Scale bar = 50 μm.

**Fig 6:**
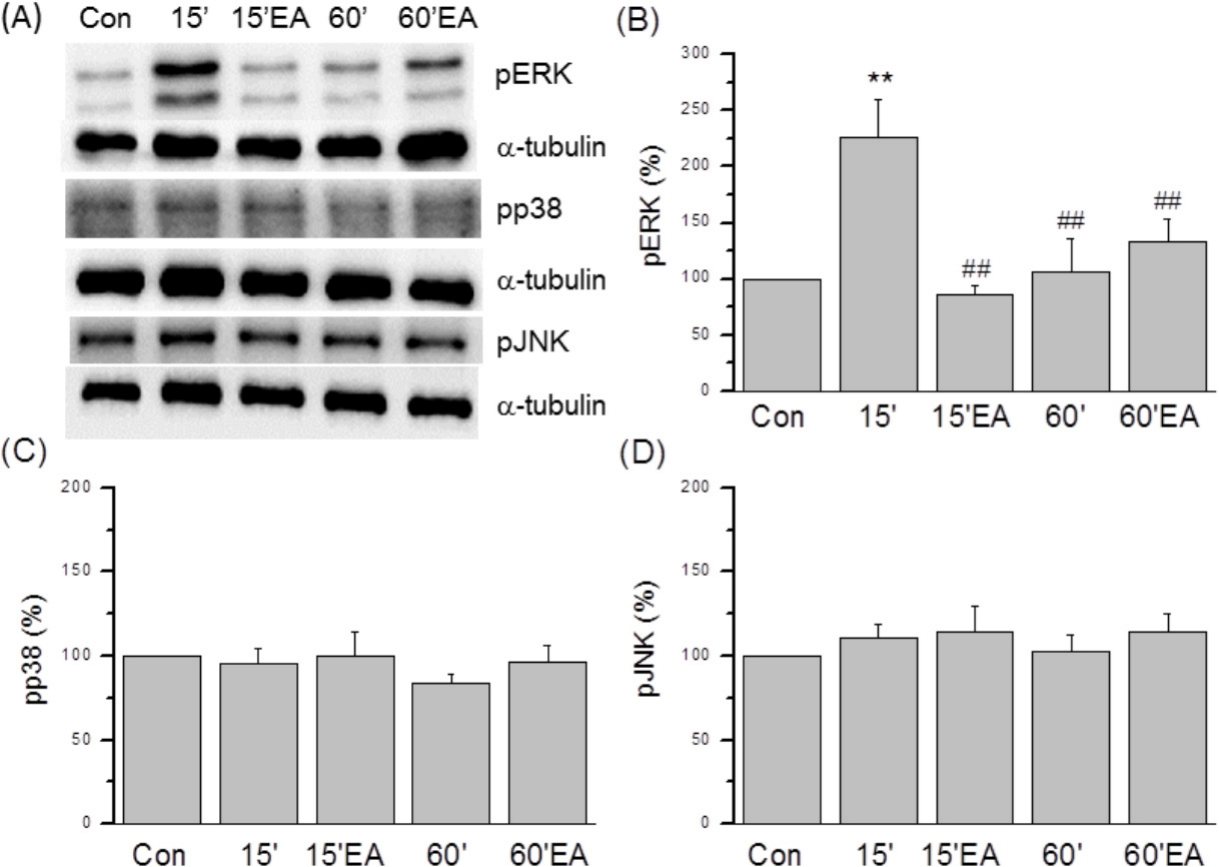
The expression of pERK, pp38, and pJNK proteins in L3-L5 DRG. (A) pERK, pp38, and pJNK kinases were measured by Western blot in lysates from DRG. (B) pERK expression was increased at 15 min after acid injection in FM mice and reversed by EA. No change in pERK expression was observed at 60 min after injection. (C) pp38 expression in DRG was not altered after acid injection and/or EA. (D) pJNK expression was not altered after acid injection and/or EA. α-tubulin expression was the internal control. Con = Control; 15 = 15 min after acid injection in FM group; 15EA = 15 min after acid injection in EA group EA. 60 = 60 min after acid injection in FM group; 60EA = 60 min after acid injection in EA group.

Increased phospho-activation of ERK (phospho-ERK, pERK) is well established in FM models, but it is not known if ERK signaling is also regulated by EA. The number of pERK-IR neurons was higher than control 15 min after the second acid injection (Fig 5B vs Fig 5A), and again this response was reversed by EA (Fig 5C). An increase in pERK was not observed 60 min after the second injection (Fig 5D–5F). These results were confirmed by Western blot analyses (Fig 6A); pERK levels were higher than control at 15 min after the second acid injection (226.1 ± 33.6%, *p* < 0.05 compared to the control group, n = 6; Fig 6B), an effect reversed by EA (85.8 ± 9.8%, *p* < 0.05 compared to the FM group, n = 6; Fig 6B), while at 60 min after FM, pERK levels did not differ significantly between FM and FM + EA groups (105.8 ± 29.8% and 133.5 ± 19.5%, *p* < 0.05 compared to the FM group, n = 6; Fig 6B).

In contrast to pERK, DRG pp38 expression was unaltered in both FM and FM + EA groups at 15 min after acid injection (95.8 ± 8.8% and 99.7 ± 15.1%, *p* > 0.05 compared to the control group, n = 6; Fig 6C) and at 60 min post-injection (84.1 ± 5.3% and 96.6 ± 9.6%, *p* > 0.05 compared to the control group, n = 6; Fig 6C). Expression of pJNK in the DRG did not differ significantly among groups (Fig 6D).

**Fig 7:**
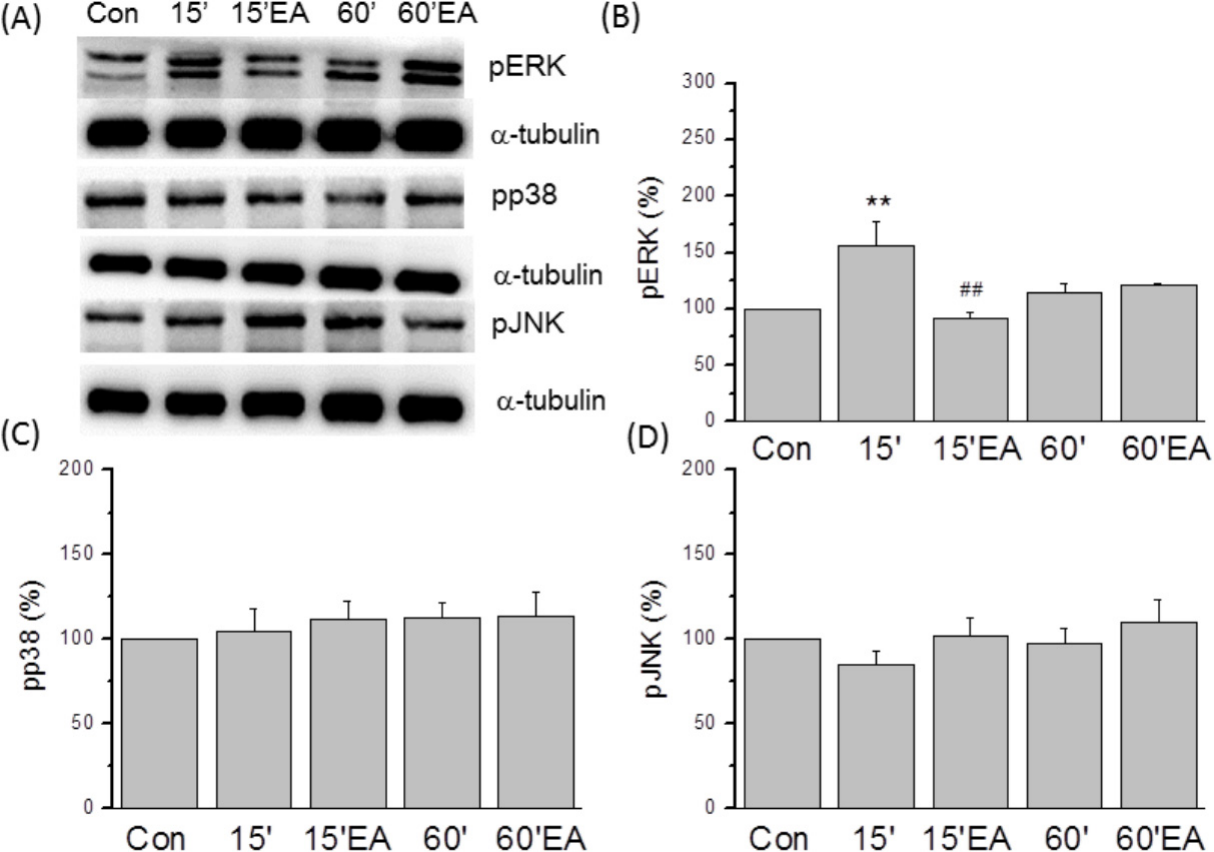
The expression of pERK, pp38, and pJNK in lumbar SC. (A) Western blots of lumbar SC lysates. (B) pERK expression was increased at 15 min after acid injection and reversed by EA. This increase in pERK was not observed at 60 min after injection. (C) pp38 expression was not altered after acid injection and/or EA. (D) pJNK was not altered after acid injection and/or EA. α-tubulin was the internal control. Con = Control; 15 = 15 min after acid injection in FM group; 15EA = 15 min after acid injection in EA group. 60 = 60 min after acid injection in FM group; 60EA = 60 min after acid injection in EA group.

We then tested if pERK, pp38, and pJNK protein levels were altered in the SC after FM modeling and EA (Fig 7A). Similar to the DRG, pERK expression was elevated in the SC of FM mice 15 min after the second acid injection (164.6 ± 33.9%, *p* < 0.05 compared to the control group, n = 6; Fig 7B) and this overexpression was reversed by EA (92.8 ± 10.6%, *p* < 0.05 compared to the FM group, n = 6; Fig 7B). At 60 min after the second acid injection, however, pERK levels were similar in FM and FM + EA groups (115.1 ± 10.5% and 116.7 ± 10.1%, *p* < 0.05 compared to the FM group, n = 6; Fig 7B). Spinal levels of pp38 were unaltered in both FM and FM + EA group mice at 15 min after acid injection (112.4 ± 18.3% and 118.6 ± 15.6%, *p* > 0.05 compared to the control group, n = 6; Fig 7C) and at 60 min post-injection (122.2 ± 9.0% and 124.7 ± 19.3%, *p* > 0.05 compared to the control group, n = 6; Fig 7C). In the spinal cord, pJNK protein expression did not differ significantly among groups (Fig 7D). These data suggest that ERK signaling is transiently activated in both DRG and SC following acid injection, leading to hyperalgesia through both peripheral and central effects on pain transmission. Moreover, reversal of TRPV1-ERK hyperactivity may account for the therapeutic effects of EA.

### Overexpression of pERK in FM mice was attenuated in TRPV1 knockout mice

**Fig 8:**
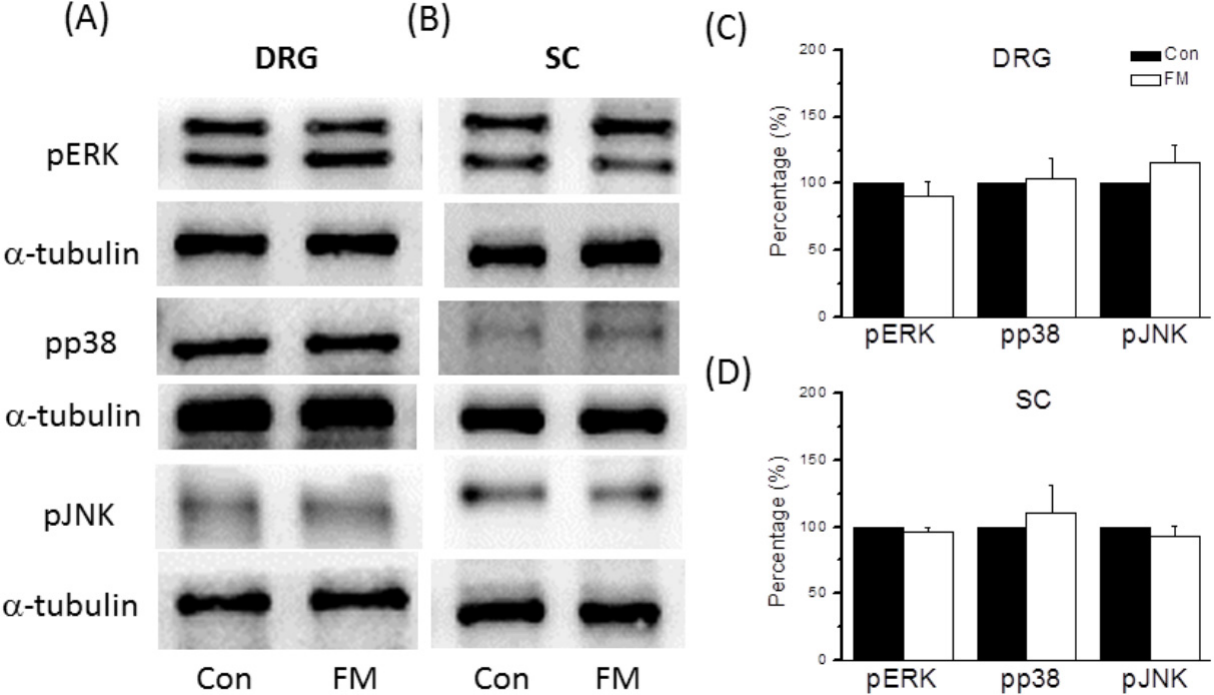
Expression levels of pERK, pp38, and pJNK in L3-L5 DRG from TRPV1 null mice. (A) Western blots of DRG lysates probed for pERK, pp38, and pJNK. (B) Expression levels of pERK, pp38, and pJNK were unchanged at 15 min after injection. α-tubulin was the internal control. Con = Control; FM = acid induced FM pain.

To provide further evidence for a causal role of TRPV1 overexpression in hyperalgesia (and EA-mediated analgesia), we examined the DRG expression levels of pERK, pp38, and pJNK in TRPV1 knockout mice (Trpv1^-/-^). In contrast to wild types, pERK levels were not increased at 15 min after repeated acid injection in Trpv1^-/-^ mice (Fig 8A, upper panel, 91.0 ± 10.4%, *p* > 0.05, n = 6). Furthermore, compared to the control group, neither pp38 nor pJNK were increased at 15 min after the second acid saline injection (Fig 8A, middle and lower upper panel, 103.2 ± 15.5% and 116.2 ± 12.4%, *p* > 0.05, n = 6). Finally, we tested whether pERK overexpression was induced in the SC by FM modeling. Expression levels of pERK, pp38, and pJNK were unchanged in SC lysates at 15 min after the second injection (Fig 8B). All results are summarized in Fig 8C and 8D.

### Involvement of TRPV1 and pERK in the induction of FM pain

**Fig 9:**
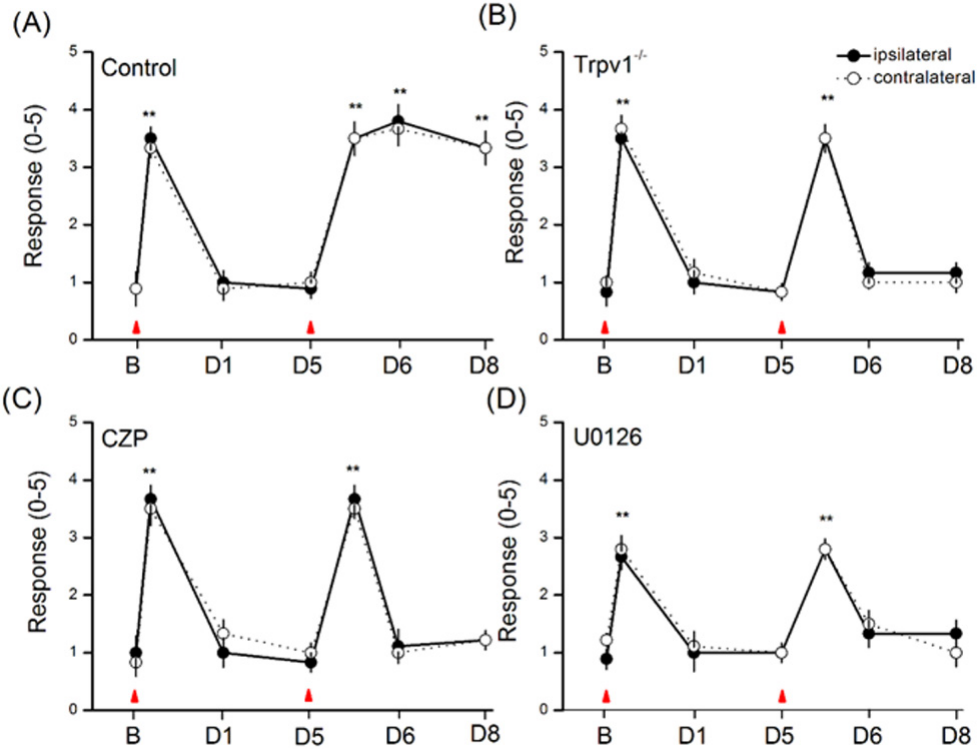
Targeting TRPV1 and ERK signaling pathway attenuated mechanical hyperalgesia by von Frey filaments. (A) Mechanical responses of FM mice. (B) Mechanical responses of Trpv1^-/-^ mice. (C) Mechanical responses of FM mice pretreated with CZP. (D) Mechanical responses of FM mice pretreated with U0126. Mice were tested before injection (baseline, B), 4 hours after injection, day 1 (D1), day 5 (D5), day 6 (D6), and day 8 (D8). Red arrowheads indicate acid injection. **p < 0.01 compared to baseline. (n = 6 mice per group).

We further tested whether deletion or inhibition of TRPV1 can influence the initiation of hyperalgesia. Dual acid injections spaced 5 days apart induced only transient hyperalgesia in Trpv1^-/-^ mice (Fig 9A and 9B, n = 8). In addition, injection of the TRPV1 antagonist capsazepine simultaneously with the two acid saline injections significantly reduced the development of mechanical hyperalgesia (Fig 9C, n = 8). Furthermore, co-injection of the ERK inhibitor U0126 with acid saline also dramatically reduced mechanical hyperalgesia (Fig 9D, n = 8). These results strongly suggest that upregulation of TRPV1 and ERK overactivation mediate mechanical hyperalgesia.

## Discussion

The contributions of TRPV1 and TRPV4 channel activity have been well documented in several pain models [30,31]. Inhibition or underexpression of TRPV1 and TRPV4 generally results in antinociceptive effects in animal models, but the role of these channels in FM is still unclear [14,20]. We describe a novel antinociceptive role of TRPV1 involving the ERK signaling pathway in an FM model. Intramuscular injection of acid saline (pH 4.0) increased the expression of TRPV1 (but not TRPV4) in DRG neurons and activated ERK. Furthermore, TRPV1 overexpression and ERK phospho-activation were reversed by 2 Hz EA at the ST36 acupoint. Notably, both TRPV1 and TRPV4 were upregulated in the spinal dorsal horn of FM mice, suggesting additional roles in central sensitization. These increases in TRPV1 and TRPV4 expression were also reversed by EA, strongly suggesting that TRPV1 and TRPV4 are involved in nociceptive plasticity and the therapeutic effects of EA.

TRPV1 can be activated by capsaicin, protons, heat, and anandamide [16,32]. TRPV1 protein expression and function are also increased by several endogenous mediators released under inflammatory conditions. TRPV1 antisense oligonucleotides or antagonists can reliably attenuate mechanical hyperalgesia. Loss of TRPV1 function in mice was associated with higher withdrawal threshold in the von Frey mechanical test, suggesting that TRPV1 upregulation is crucial for mechanical hyperalgesia [33–35]. Indeed, under pathological conditions TRPV1 can be activated at normal physiological temperature and by only mild acidosis. In addition, migraine hyperalgesia is associated with reduced TRPV1 activation threshold [36]. Ro *et al*., reported that intramuscular injection of capsaicin (a TRPV1 agonist) and mustard oil (a TRPA1 agonist) induced mechanical hyperalgesia, which could be abolished by pretreatment with specific antagonists [37]. These results suggest that blockade of TRPV1 channels leads to a reduced nociceptive response. Lund et al. reported that action potential parameters were changed after acid saline injection with similar time course as behavioral changes [38]. They also found that TRPV1 was colocalized with metabotropic glutamate receptors (mGluRs) and that mGluR antagonist injection prevented capsaicin-induced nociceptive behaviors [39]. Recently, TRPV1 was reported to be highly expressed at the BL40 acupoint, implicating this channel in EA-mediated analgesia [40]. Our results indicated that TRPV1 is upregulated in both the DRG and dorsal horn of an FM model and that overexpression can be ameliorated by 2-Hz EA at the ST36 acupoint, strongly suggesting that the analgesic effect of EA is associated with TRPV1 downregulation.

TRPV4 is involved in sensing osmolarity, mechanical force, and heat. Mechanical hyperalgesia is attenuated by spinal application of TRPV4 antisense oligonucleotides or by TRPV4 gene depletion [24,41,42]. Wei *et al*., reported that TRPV4 is crucial for tension-mediated migraine headache [43]. In addition, TRPV4 may be a key mediator in paclitaxel chemotherapy-induced neuropathic pain [44], and in pancreatitis pain [45]. Moreover, thermal pain induced by chronic compression of DRG neurons involves activation of the TRPV4-NO-cGMP-PKG pathway [14]. A recent study reported that TRPV4 plays an important role in inflammatory mediator-induced hyperalgesia through cAMP, PKA, and PKCε. TPRV4 can be sensitized by prostaglandin E2 (PGE2), serotonin, and PAR2. Grant *et al*., demonstrated that inflammation induced PLCβ, PKA, PKC, and PKD signaling in the dorsal horn, leading to CGRP and SP release and activation of TRPV4 [23]. However, TRPV4 was not altered in the DRG of our FM model mice, suggesting that it does not participate in the peripheral hyperalgesia, characteristic of this model. Alternatively, TRPV4 was upregulated in the spinal cord, suggesting a role in central pain sensitization.

Chronic pain is often defined as a long-term hypersensitivity of peripheral nociceptive neurons with ensuing central sensitization. Increased activation of pERK has been reported in the central amygdala and the paraventricular thalamic nucleus of FM models [1,8]. Activation of ERK enhances synaptic transmission in the central amygdala, accounting for central sensitization and behavior hypersensitivity. The increased ERK activity in the acid-induced FM pain model can be prevented by Ca_V_3.2 gene deletion, indicating an important contribution by the Ca_V_3.2-ERK signal pathway [1]. Furthermore, the μ-opioid receptor agonist morphine, as well as glutamate receptor antagonists, K^+^ channel openers, and Na^+^ channel blockers can reliably alleviate FM-like pain in animal models. In contrast, anticonvulsant drugs, cyclooxygenase-2 (COX-2) inhibitors, and the benzodiazepine diazepam did not reduce FM-induced mechanical hyperalgesia [46]. These results suggest a central role for acid-sensitive pain-related receptors/channels in hyperalgesia in the absence of inflammation.

Sluka *et al*., first reported that FM could be reduced in mice lacking ASIC3 gene expression [47]. Our previous publication showed that loss of SP release initiates FM-induced mechanical hyperalgesia [3]. Chen et al. suggested that FM-mediated mechanical hyperalgesia could be prevented by injection of antagonists of ASIC3 and TRPV1 [2]. They also found that acid could activate receptors or ion channels in nerve terminals of GM muscle (without ASIC3 or TRPV1 expression) to release SP. Much is known about how ion channels give rise to peripheral sensation and central sensitization, leading to FM pain, but less is known about the signaling pathways involved in FM pain. In the current study, we showed that pERK, but not pp38 and pJNK, was increased during a critical period after the second acid saline injection. The increase in pERK was reduced by EA. Experiments with TRPV1 knockout mice also indicated a crucial role for these channels in FM induction. Moreover, knockdown of TRPV1 prevented the acid-induced increase in pERK expression. We suggest that mice lacking TRPV1 channels cannot sense acidosis at peripheral nerve terminals. In the absence of this sensation, pathways leading to peripheral and central sensitization are not activated.

Manjavachi et al. demonstrated that intramuscular injection of selective ERK, p38, or JNK inhibitors significantly reduced IL-6-mediated muscle pain. They further used flow cytometer to confirm that pERK, p38, and JNK were simultaneously phosphorylated at 5 min after IL-6 injection [48]. In mice with visceral pain, p38 was increased by TRPA1 receptors [49]. Injection of CFA into the hindpaw activated JNK in DRG neurons. Furthermore, treatment with a JNK inhibitor reliably alleviated thermal hyperalgesia. Zhuang et al., concluded that activation of JNK in the DRG and spinal cord is critical for expression of neuropathic pain in rats [50]. In the present study, however, only pERK was phosphorylated (activated) 15 min after acid saline injection, while p38 and JNK were not. We suggest that pERK but not p38 or JNK are involved in induction of hyperalgesia in this FM model.

We concluded that mechanical hyperalgesia in FM model mice was mitigated by EA at the ST36 acupoint. Hyperalgesia was associated with DRG neuron hyperexcitability, TRPV1 overexpression, and ERK activation. Increased TRPV1 and pERK may further induce central sensitization, resulting in bilateral mechanical hyperalgesia. Upregulation of TRPV1, TRPV4, and pERK may be involved in spinal central sensitization as well and these responses were reversed by EA. Results showing lack of mechanical hyperalgesia in TRPV1 null mice and mice injected with TRPV1 and ERK inhibitors strongly suggest that TRPV1 overexpression is necessary for the development of this FM-like pain state.
